# The Units of General Hospital Construction

**Published:** 1907-06-08

**Authors:** 


### HOSPITAL ADMINISTRATION.

### CONSTRUCTION AND ECONOMICS.

### THE UNITS OF GENERAL HOSPITAL CONSTRUCTION.

#### THE NURSING AND WARDMAIDS' STAFF FOR EACH UNIT.

Although a hospital is constructed on the ward unit system, it does not necessarily follow that the cost of administration per bed, or per patient, should be greater than one worked on the old principle, where patients of different members of the staff are treated in the same wards. In practice it is found to be much more satisfactory and more economical to assign a separate unit to each member of the visiting staff. Under this system the exact cost of each of the units can be compared, the number and nature of the cases and operations noted.

With regard to the staffing of the ward units of a general hospital, there are many points to be considered. A resident assistant should be allocated to each unit to devote his undivided attention to the patients in the wards. On no account should he be called upon to attend the private operations of the visiting surgeon, or act as his assistant outside the hospital. This either takes him away from his proper duties or deprives him of the necessary outdoor exercise which helps to keep him fit for his hospital work, and he takes the place, without in some cases the usual fee for such assistants, of a young graduate beginning private practice, who has the time to devote to such work.

The proportion of nurses to patients varies considerably in different countries, in different hospitals in the same country, and even in different units of the same hospital. In preparing plans for a hospital the architect is at once met with the difficulty that no standard has yet been fixed giving the number of nurses and the accommodation to be provided for them in proportion to the number of patients. In every hospital there is what should be regarded as the permanent nursing staff. In addition, there is the emergency staff, and allowance must be made for a number of nurses being on holiday and a few on the sick list. In fixing, then, the number of nurses in proportion to the number of patients, all these factors must be considered.

Further, in calculating the number of nurses required for a hospital with a certain number of beds, note must also be taken of the number of nurses engaged in the casualty and out-patient departments. It is claimed for some hospitals that the number of nurses engaged in their out-patient department is very large in proportion to the number of their occupied beds. This is a matter that can be easily stated, and due allowance can be made in making any calculation of the total number of nurses required per occupied bed. Other hospitals claim that their nurses have shorter hours and longer holidays, and in consequence their nursing staff is greater in proportion to the number of occupied beds, as compared with other hospitals. But in dealing with this question we must not only consider the number of nurses required in proportion to the number of occupied beds, but must also find out the proportions of trained nurses, nurses and probationers. In some hospitals, for example, the probationers all pay a premium and receive no salary; in others a nurse is considered fully qualified after two years' training. In most hospitals the proportion of first year probationers to nurses is about 28 per cent. It is therefore important to know exactly the total number of nurses employed, the number in the different years of training, and the number fully trained, for if we were to draw conclusions from the salaries paid to the nursing staff as a whole, it might be found that some hospitals have a larger proportion of probationers to nurses than others, and as a result the cost per bed for nursing is much smaller than where the total number on the nursing staff is the same, but where the staff is more experienced and more highly paid. It must be evident, therefore, that in dealing with the efficiency of the nursing staff, the total number on the staff and the sum paid for nursing is not enough. We must have details of how the staff is constituted and the relative proportion of first-year probationers to nurses. In no case should such probationers for the purposes of the work in the hospital constitute more than one-third of the staff. In old buildings where the wards are small and scattered, a larger proportion of nurses is required than in a modern hospital constructed and administered on the complete unit principle. But if properly constructed and administered, and *no unreasonable demands are made*, the proportion of one nurse to every three patients can be regarded as a fair workable standard. In constructing a modern hospital, therefore, an architect should make provision in the nurses' home for a separate bedroom for one nurse to every three occupied beds in the hospital. He should, however, provide some extra bedrooms, say 7 per cent. of the total, or 40 bedrooms as a maximum for every 100 occupied beds.

The whole nursing staff is under the direct supervision of the matron, and a permanent staff is assigned to each of the different units. A ward sister is placed in charge of each unit, and she is directly responsible to the matron for the efficient nursing of the patients. An emergency nurse should only be asked for when there is special urgency; and in making requisition for an extra nurse, the sister should inform the matron of the particular patient to whom the nurse is to be assigned and the special nature of the urgency. When the urgency is over the sister should inform the matron, the nurse should be relieved, and be available to take duty elsewhere. The ward sister should see that the instructions of the physician or surgeon, as the case may be, are faithfully carried out by the nurses placed under her supervision, clearly bearing in mind at all times that she is a servant of the institution and directly responsible to the matron for her own work as well as that of the nurses under her charge. Besides attending to the proper nursing of the patients, the ward sister is responsible for the thorough cleanliness of her wards and their annexes, for the inventory of the crockery, linen, and other furnishings of the unit, and should be able to give an accurate account to the matron of every article worn out, broken, or lost. While the treatment of the patients is her first consideration, no sister can be regarded as efficient who neglects the proper supervision and training of her nurses and wardmaids, for unless the work of the nurses is carefully supervised and the wards and their annexes are kept scrupulously clean, the treatment of the patients cannot be satisfactory. For the economical administration of a ward unit the sister should be supplied with an inventory of all the crockery, cutlery, etc., in each ward. An accurate record should be kept of every article which has been broken or lost and the number of articles replaced. This should be checked by the matron or her assistant once a month.

The following shows how this can be systematically carried out:—

month. We are glad to note that a marked economy in the use of surgical dressings is now being shown in the best administered hospitals. We are confident that every institution which uses in each surgical ward the dressings book referred to will find it a great aid to reduction of expense in these expensive materials. All these requisitions should be signed daily by the superintendent. The keeping of such a record serves a double purpose. An administrator knowing exactly the number of patients who have been treated, the number of operations performed, the amount and cost of the surgical dressings and other materials used, is in a position to place before his managers an accurate comparative statement from day to day, week to week, or month to month, of the different units of the hospital, and show the results. The surgeon in charge of the unit, if he desires, can see any day what it costs to treat his patients. He can also compare day with day or week with week, and he may find this of great value in the treatment of his private patients. In some cases demands are made in hospitals for expensive fittings and appliances which are not to be found in private nursing homes.

### The Wardmaids.

Having dealt with the number of nurses to be allowed for a hospital of a known number of beds,

*Sister's Monthly Return of Daily Store Issues and Breakages.*

The ward sister should be supplied with a further inventory of the stock of linen, etc., in the wards. At the end of each week articles worn out should be noted and a record made of new articles supplied to take their place. By means of these simple registers the efficiency or otherwise of a sister as regards her supervision of the wards can be easily estimated. Every detail of expenditure in a ward unit can be compared with those of any other unit in the hospital, leakages can be readily detected, and waste and extravagance can be checked. In addition to these books the sister in charge of a surgical ward unit should have a daily record of all the surgical dressings and appliances, such as clinical thermometers got for ward use, and by adopting the "Surgical Dressings" book, which may be obtained from The Scientific Press, as part of the uniform system for each ward, it is possible to note the quantity of each of the articles used daily for each and stated the proportion of one nurse to every three patients as a reasonable provision, including day and night service, we must now consider the number of wardmaids required in a modern hospital to perform the menial duties, which in former days had to be carried out by the nurse. As a general rule a wardmaid should be allowed for each ward, and, in addition, such assistance as the matron considers necessary for the cleaning of corridors and stairs leading to the unit. The number of nurses required for a ward unit must vary according to the nature and severity of the cases, but this does not apply to the number of wardmaids. In a medical unit such as has been described and illustrated a wardmaid should be allowed for each ward. And in the surgical unit, besides a wardmaid for each ward, an additional maid should be granted for the operating theatre and the rooms connected with it.

